# QT Interval Prolongation Associated with Intramuscular Ziprasidone in Chinese Patients: A Case Report and a Comprehensive Literature Review with Meta-Analysis

**DOI:** 10.1155/2014/489493

**Published:** 2014-11-04

**Authors:** Xian-Bin Li, Yi-Lang Tang, Wei Zheng, Chuan-Yue Wang, Jose de Leon

**Affiliations:** ^1^Beijing Key Laboratory of Mental Disorders, Department of Psychiatry, Beijing Anding Hospital, Capital Medical University, No. 5 Ankang Lane, Dewai Avenue, Xicheng District, Beijing 100088, China; ^2^Center of Schizophrenia, Beijing Institute for Brain Disorders, Laboratory of Brain Disorders (Capital Medical University), Ministry of Science and Technology, Beijing 100088, China; ^3^Department of Psychiatry and Behavioral Sciences, Emory University School of Medicine, Atlanta, GA 30322, USA; ^4^Mental Health Research Center, Eastern State Hospital, University of Kentucky, Lexington, KY 40511, USA; ^5^Psychiatry and Neurosciences Research Group (CTS-549), Institute of Neurosciences, University of Granada, 18071 Granada, Spain; ^6^Biomedical Research Centre in Mental Health Net (CIBERSAM), Santiago Apóstol Hospital, University of the Basque Country, 01004 Vitoria, Spain

## Abstract

Intramuscular (IM) ziprasidone has been associated with QTc interval prolongations in patients with preexisting risk factors. A 23-year-old male Chinese schizophrenia patient experienced an increase of QTc interval of 83 milliseconds (ms) after receiving 20 mg IM ziprasidone (baseline and increased QT/QTc were, respectively, 384/418 and 450/501). This was rated as a probable adverse drug reaction (ADR) by the Liverpool ADR causality assessment tool. A systematic review including all types of trials reporting the effect of IM ziprasidone on the QTc interval prolongation identified 19 trials with a total of 1428 patients. Mean QTc change from baseline to end of each study was −3.7 to 12.8 ms after IM ziprasidone. Four randomized trials (3 of 4 published in Chinese) were used to calculate a meta-analysis of QTc interval prolongation which showed no significant differences between IM ziprasidone and IM haloperidol groups (risk ratio 0.49 to 4.31, 95% confidence interval 0.09 to 19.68, *P* = 0.06 to 0.41). However, our review included two cases of patients who experienced symptoms probably related to QTc prolongation after IM ziprasidone. Thus, careful screening and close monitoring, including baseline ECG, should be considered in patients receiving IM ziprasidone for the first time.

## 1. Introduction

The second-generation antipsychotic ziprasidone is available for intramuscular (IM) use in the management of acute agitation associated with schizophrenia [1]. IM ziprasidone is described as potentially causing QT interval prolongation in large doses or in patients with other risks for interval prolongation [2]. Other antipsychotics, such as sertindole and thioridazine, prolong the QT interval and are associated with cases of torsades de pointes and sudden death [3]. The clinical significance of QTc prolongation induced by ziprasidone administration is unclear [4]. In fact, reports of cardiac adverse drug reactions (ADRs) that are clearly associated with ziprasidone remain scarce [5]. A recent pharmacoepidemiology study suggested that ziprasidone and amisulpride had potential torsadogenic risk similar to haloperidol [6].

In China, IM ziprasidone is the first and only atypical antipsychotic agent in clinical use for agitation associated with schizophrenia. Although previous studies have demonstrated that IM ziprasidone is well-tolerated for treatment of agitation in schizophrenia in Western populations [1], clinicians now recognize that racial differences may contribute to altered tolerability [7]. The data on efficacy and safety of IM ziprasidone for agitation are still scarce in China, especially the effect of IM ziprasidone on QTc interval [7]. Here we present a Chinese patient with no known risk factors who experienced a prolonged QT interval after receiving a modest dose of IM ziprasidone.

## 2. Case Presentation

Mr. A, a 23-year-old Chinese male, was brought in by his family to the emergency department (ED) with acute psychotic symptoms including disorganized speech, delusion, and agitation. There was no history of substance abuse. A provisional diagnosis of schizophrenia was made. He had never taken antipsychotics and had no preexisting cardiovascular conditions and no known medical illness. Physical examination was unremarkable. His weight was 68 kg and his body mass index (BMI) was 22.2. The basic metabolic panel values, including serum potassium and glucose levels, were all within normal limits. Serum magnesium was not measured but he had no clinical condition associated with hypomagnesemia. Although psychotic symptoms were prominent at admission, his vital signs remained relatively stable at arrival: blood pressure (BP): 100/70 mmHg; pulse: 76 beats per minute (bpm); respiration rate: 18 respirations/minute; and temperature 36.0°C. A baseline electrocardiogram (ECG) was obtained which showed normal sinus rhythm and a QT/QTc of 384/418 milliseconds (ms) at a pulse of 78 bpm.

During the ED stay, an initial IM ziprasidone dose of 10 mg was given and then IM doses of 10 mg with oral oxazepam of 30 mg were prescribed every 6 hours as needed for agitation. The total IM ziprasidone dose was 20 mg. An ECG obtained 24 hours later showed a QT/QTc of 450/501 ms with a pulse rate of 94 bpm. The increased QTc from baseline was 83 ms, a significant prolongation, but no symptoms occurred. Ziprasidone was then switched to oral olanzapine 5 mg/day, with oxazepam 30 mg/day. His QT/QTc returned to 366/416 ms at a pulse of 93 bpm 48 hours after the last ziprasidone injection. The QT/QTc returned to 382/402 ms 72 hours after the last dose of ziprasidone. This QTc of 402 ms is 16 ms lower than at baseline and 99 ms lower than during ziprasidone treatment. The patient was later transferred to inpatient services for further treatment.

All authors agreed that this was a probable ADR, according to the Liverpool ADR causality assessment tool [8]. Therefore, this case demonstrated a significantly prolonged QT/QTc interval after modest doses of ziprasidone.

## 3. Discussion

### 3.1. Comprehensive Literature Review and Meta-Analysis

A systematic review on this topic was conducted. Our protocol of reviewing IM ziprasidone for acute psychosis has been published online (http://www.crd.york.ac.uk/prospero/). The registration number was CRD42014007542 at the Preferred Reporting Items for Systematic Reviews and Meta-Analyses (PRISMA), which provides an evidence-based minimum set of items for reporting in systematic reviews and meta-analyses.

All types of trials that included the effect of IM ziprasidone on the QTc interval prolongation were eligible for inclusion. We included case series, retrospective studies, open-label prospective trials, and randomized controlled trials (RCTs). We excluded meta-analyses and systematic reviews. We searched the PubMed, Embase, and Cochrane Library databases. We also searched the Chinese databases (CBM and CNKI databases) using the same key words. The search included all studies published between January 2000 and December 2012, regardless of language. The keywords used for the searches included schizophrenia, intramuscular ziprasidone, QTc, and agitation. The keywords were used in combination with the Boolean operators AND, OR, and NOT. We used the “related article” function to supplement the research. We also manually searched bibliographies of RCTs, meta-analyses, case reports, and systematic reviews for studies that were missed in the initial electronic search.

Review authors X-BL and WZ considered all included studies initially, without seeing comparison data, to judge clinical, methodological, and statistical heterogeneity, which provided the basis for deciding whether each study would be included for meta-analysis or other data synthesis. Data from all included studies was extracted and organized into standard, simple forms. In addition, to ensure reliability, X-BL independently extracted data from a random sample of these studies, comprising 30% of the total. Data presented only in graphs and figures was extracted whenever possible but was included only if two authors independently had the same result. We also attempted to contact authors through an open-ended request in order to obtain missing information or for clarification whenever deemed necessary.

The meta-analysis was performed according to the recommendations of the Cochrane Collaboration, using the Review Manager Version 5.1.7.0 software. Two authors (X-BL and WZ) independently extracted data for analysis. We presented the summary statistic of dichotomous outcomes as a risk ratio for QTc interval prolongation ≥ 450 ms, 450 ms ≤ QTc interval < 480 ms, and QTc interval ≥ 480 ms. The Mantel-Haenszel method was used to combine the summary statistic. We used I2 methods to assess statistical heterogeneity. We used a fixed effect model if no heterogeneity existed or I2 < 50% [24]. All statistical differences were considered significant when *P* < 0.05.

**Figure 1:**
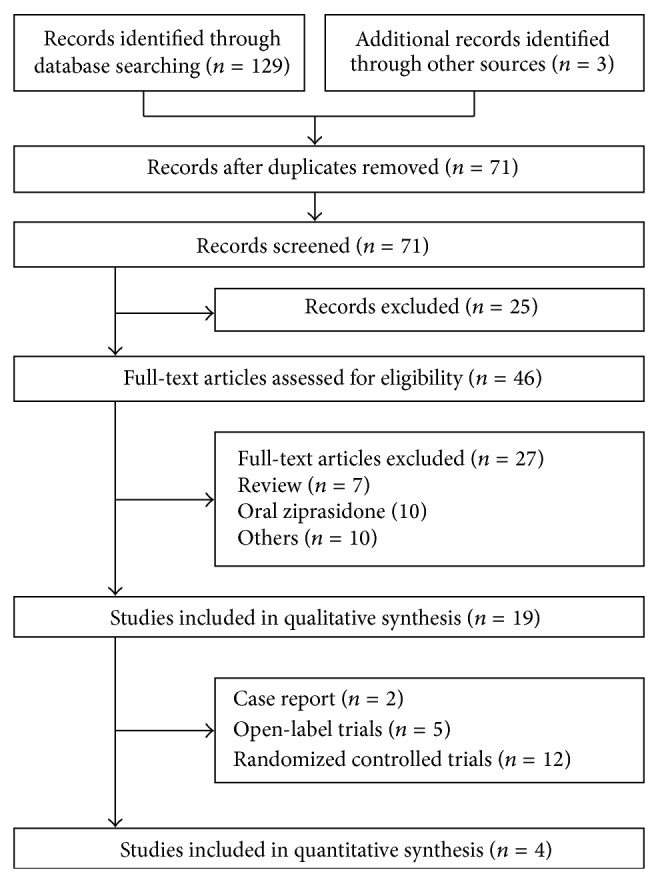
PRISMA flow diagram.

**Figure 2:**
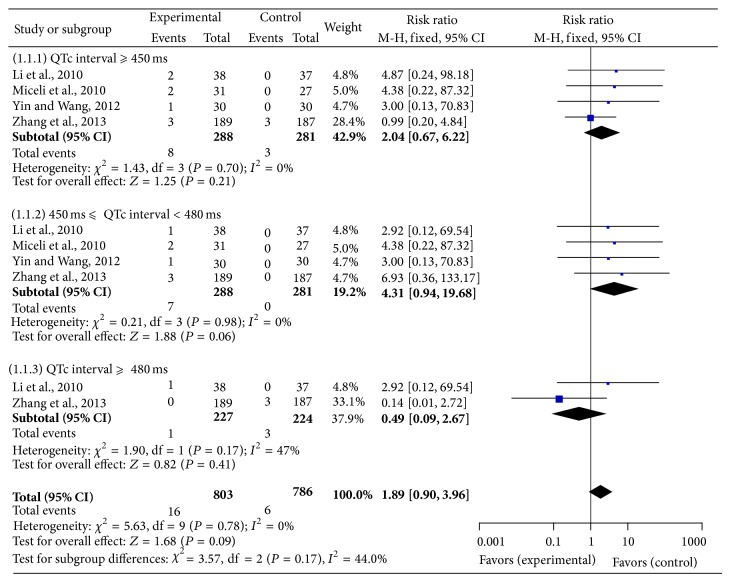
Meta-analysis comparing QTc in patients with intramuscular ziprasidone versus haloperidol.

The search yielded 132 articles, 71 records remained after duplicates were removed, 46 full-text publications were assessed for eligibility, and finally 19 articles were included in qualitative synthesis (Figure 1). The decision to include a study or not in the meta-analysis was based on judgments about clinical and methodological issues, and statistical heterogeneity; 4 studies were included in the quantitative synthesis. In total, 1428 patients had received IM ziprasidone treatment and 836 patients had received IM haloperidol treatment for agitation. The 19 reports included 12 RCTs (ziprasidone versus haloperidol) [1, 7, 9–12, 14–16, 19, 22, 25], 5 open-label perspective trials [13, 17, 18, 20, 21], and 2 individual case reports [2, 4]. After multiple discussions among the authors, it became clear that one trial had two studies [18] but one of them was also reported in a later larger study [19]; therefore, one of these two studies was excluded from Table 1, which summarizes the characteristics of the included 19 studies. In only one of the studies [7] was it possible to estimate the effect size of the mean difference (see Table 1, footnote 5) using Cohen's method [23]. Four studies [7, 16, 19, 22] with 288 patients, including three published in the Chinese language [7, 16, 22], were included in our meta-analysis (Figure 2).

In summary, in the IM ziprasidone group, only three subjects (0.2%) had a QTc ≥ 500 ms; 3 subjects (0.2%) reached a QTc ≥ 480 ms; 10 (0.7%) had a QTc ≥ 450 ms. On the other hand, within the haloperidol group, one subject had a QTc ≥ 500 ms, 3 reached a QTc ≥ 480 ms, and 3 had a QTc ≥ 450 ms. Seven patients (0.4%) of the IM ZPD group had QTc changes that exceeded 60 ms relative to the time-matched baseline values. By combining all the studies included in Table 1, the mean change in QTc from baseline to end of study was −3.7 to 12.8 ms in the ziprasidone group; the corresponding QTc change in the haloperidol group was −3.5 to 14.7 ms.

Overall, IM ziprasidone had a mild effect on the QTc interval prolongation. First, ziprasidone was associated with modest concentration-related QTc increases [20]. Second, an open-label perspective trial showed similar results including (a) one with only 3 subjects having >60 ms prolonged QTc interval [17] and (b) another [13] indicating that IM ziprasidone did not appear to influence atrial and ventricular electrical conduction in drug-free inpatients with schizophrenia. However, the diagnosis of schizophrenia might influence atrial conduction and even be associated with atrial fibrillation. Third, the ziprasidone versus haloperidol RCTs showed that both agents were generally well tolerated; few subjects experienced significant QTc interval prolongation in the ziprasidone group (Table 1).

We paid particular attention to cardiac ADRs that may be linked to IM ziprasidone. In the prospective trials and RCTs, no patients reported symptoms related to QTc prolongation. Two cases from other studies did have symptoms associated with prolonged QTc interval. One patient lost consciousness within 45 min of IM ziprasidone injection of 20 mg; she was pulseless and hypotensive and her physical examination was remarkable for absent pulses and signs of poor perfusion [4]. ECG monitoring demonstrated bradycardia (30–40 beats per minute) with a third-degree heart block. She had spontaneously converted to a normal sinus rhythm after a 36-hour rescue including cardiopulmonary resuscitation. After using an ADR scale the authors [4] concluded it was a probable ziprasidone-induced ADR since the patient was not taking any other medication and it was not an obvious heart-related illness but, as the patient was 70 years old with dementia and hypertension, they acknowledge that other unknown medical conditions or risks might have also contributed. The other subject was a 47-year-old male who admitted using crack cocaine 72 hours before the presentation. He had palpitations and weakness 45 minutes after receiving ziprasidone 20 mg IM [2]. His maximum QT/QTc interval was 612/517 milliseconds. Sanaei-Zadeh [26], commenting on this case, emphasized that the baseline ECG before administering ziprasidone was already prolonged with a QT/QTc of 484/475 milliseconds at a pulse of 58 beats per minute.

A meta-analysis of QTc interval prolongation ≥ 450 ms, 450 ms ≤ QTc interval < 480 ms, and QTc interval ≥ 480 ms showed no significant differences in the IM ZPD group compared with the haloperidol group, as demonstrated by the Mantel-Haenszel fixed risk ratio, 0.49 to 4.31 (95% confidence interval 0.09 to 19.68; I2 = 0%, *P* = 0.06 to 0.41) (Figure 2).

### 3.2. Case Discussion

This patient, a young, healthy male with no preexisting cardiac conditions, received only the recommended dose of IM ziprasidone for acute agitation (20 mg IM). The only concurrent use of medication was oxazepam, which has no known effects on the QT interval [27]. However, the change in QTc from baseline was 83 ms, and his maximum QT/QTc interval was 450/501 ms.

While QT/QTc prolongation has been reported in patients receiving IM ziprasidone, the extent of prolongation in this patient is perhaps among the greatest in China, especially considering that this occurred in a young, healthy patient with no known preexisting conditions. Some unknown factors, including genetic defects at the heart potassium ion channel, may have played a role [28, 29]. Ziprasidone is only partly metabolized by CYP3A4 [30]; therefore, no known pharmacokinetic gene abnormalities are expected to play a role in this case.

Our subsequent systematic review found that, among 1428 patients, only three subjects (0.2%) had a QTc ≥ 500 ms. In our calculations, the mean change in QTc from baseline to end of study was −3.7 to 12.8 ms after the ziprasidone injection. This is comparable to other less comprehensive reviews such as the one completed by Camm et al. [31]. Our systematic review, including all IM trials, found more patients who experienced QTc ≥ 480 ms (3/1440 versus 1/4306) than did the study by Camm et al. (which primarily focused on oral ziprasidone). The greater IM versus oral effects may be explained by higher peak serum concentrations in IM administration [4].

Furthermore, meta-analysis of QTc interval prolongation of four RCTs, including three only published in Chinese, showed no significant differences in the IM ziprasidone group compared with the haloperidol group. However, similarity to haloperidol does not guarantee safety, as the US IM haloperidol prescribing information has a warning about torsades de pointes [32].

Changes from baseline QTc interval were clinically modest with both drugs, which is comparable to an important RCT which showed that both ziprasidone and haloperidol were generally well tolerated [19]. In general, these data are consistent with results from ziprasidone clinical pharmacology studies and other reviews focused on QTc prolongation. Taken together, they provide the most comprehensive evidence published to date that IM ziprasidone appears to be relatively safe for agitation.

Although several patients experienced significant QTc interval prolongation in the current review, no cardiac ADRs were reported in the prospective trials and RCTs. Nevertheless, our review found two cases [2, 11]; both had reported symptoms related to QTc prolongation.

Most reported cases of prolonged QT interval associated with ziprasidone use have occurred in patients with preexisting cardiovascular conditions, hypokalemia, hypomagnesemia, concurrent use of other QT prolonging medications, higher doses of ziprasidone (>40 mg IM), rapid titration of the dosage, or repeated dosing [2, 12]. Fortunately, most patients experienced no symptoms associated with the QT interval prolongation.

Most cases of drug-induced torsades de pointes occur in the context of substantial prolongation of the QTc interval, typically to values >500 ms, but QTc alone is a relatively poor predictor of arrhythmic risk in any individual patient. Some drugs that substantially prolong the QTc interval produce very low rates of torsades de pointes while others have much smaller QTc effects but are considerably more proarrhythmic [30].

### 3.3. Limitations

Although all authors agreed that this was a probable ziprasidone-induced ADR, there is no absolute proof that it was. One could argue that the QTc prolongation was an artifact, but this conclusion does not appear likely since the patient had 3 ECGs using the same equipment and no other patient at that time showed similar QTc prolongations.

Significant heterogeneity of the results in QTc ≥ 480 ms was seen in this meta-analysis, suggesting the effect of relevant moderator and mediator variables. This may be because meta- analyses combine results from trials that differ in their methodology, sample size and year, patient and treatment selection, and outcome variables. This meta-analysis only included 288 patients from four RCTs because these were the only ones identified in our search. The sample size was relatively small for meta-analysis, which prevented further data exploration. However, we included well-defined RCTs comparing ziprasidone with haloperidol. Although there were about 12 published RCTs, only 4 trials were included in this meta-analysis after a more strict quality assessment.

## 4. Conclusion

As the first and only IM atypical antipsychotic medication available in China, IM ziprasidone offers some advantages over haloperidol [7, 9], which remains the most commonly used medication for agitation in China [7]. Our case report contributes to the current literature on ziprasidone use in the Chinese population and indicates that, even in healthy patients with no preexisting conditions, a modest dose of IM ziprasidone can also cause significant prolonged QT interval. In light of reports that a prolonged QTc interval may be associated with torsades de pointes and sudden death [6], a careful screening and close monitoring including regular ECG should be considered. We were fortunate enough to get a baseline ECG that demonstrated an increase in QTc interval but we acknowledge that it is not always easy to get a baseline ECG in an agitated psychotic patient.

## Figures and Tables

**Table 1:** Summary of IM ziprasidone trials.

Study	Design	Subjects (*n*)	Dose^1^	Main findings
Brook et al., 2000 [9]	International multicenter RCT (IM ZIP versus HAL^2^) 3 d IM then 4 d PO	(90 versus 42)	10–80	No pt in any group had QTc >500 ms ZIP mean QTc change^3^ in ms: 2.14 (versus 2.22 HAL)

Brook et al., 2005 [1]	International multicenter RCT (IM ZIP versus HAL^2^) 3 d IM then 6 wk PO	(427 versus 138)	10–20	No pt in any group had QTc >500 ms Mean ZIP endpoint QTc in ms: 389.9 (versus 383.7 HAL) Mean ZIP QTc change^2^ in ms: 3.2 (versus −3.5 HAL)

Chen et al., 2010^3^ [10]	7 d open parallel RCT (IM ZIP versus HAL^2^)	(40 versus 40)	10–60	1 ZIP pt withdrew after a QTc prolongation

Daniel et al., 2001 [11]	24 h double-blind RCT	(38 versus 41)	2, 20	Mean QTc change^3^ in ms: 3.5 (2 mg) and −1.3 (20) Maximum QTc recorded was 475 ms

Daniel et al., 2004 [12]	Multicenter RCT (3 IM ZIP doses versus HAL^2^) 3 d IM, then 4 d PO	(69, 71, 66, 100 HAL)	20, 40, 80	No pt in any group had QTc >500 ms Mean QTc change^3^ in ms: 0.7 (20 mg), −2.9 (40), and 0.5 (80); −0.5 (HAL)

Emul et al., 2009 [13]	Open-label prospective trial (schizophrenia versus healthy controls; baseline versus 1.4–2 h after IM ZIP	(11 versus 11)	20	IM ZIP did not appear to influence atrial and ventricular electrical conduction in drug-free schizophrenia inpts; schizophrenia may affect atrial conduction resulting in atrial fibrillation

Jiang et al., 2008^4^ [14]	3 d open parallel-group RCT (IM ZIP versus HAL^4^)	(36 versus 35)	10–40	No pt in any group had QTc >500 ms

Lesem et al., 2001 [15]	24 h double-blind RCT	(54 versus 63)	2, 10	Mean QTc change^3^ in ms: −3.7 (2 mg) and −1.8 (10)

Li et al., 2010^4^ [16]	5 d double-blind parallel-group RTC (IM ZIP versus HAL^2^)	(38 versus 37)	10–30	3 pts experienced QTc prolongation (ms): 430, 450 and, 510

Mautone and Scarone, 2011 [17]	Phase IIIb noncomparative(3 d IM then PO for 8 w)	(150)	10	Mean QTc change^3^ in ms in 3 pts: 110 (2 d), 70 (5 d), and 55 (10 d). 2 pts discontinued due to QTc prolongation

Micelli et al., 2005 [18]	Phase I study in healthy volunteers: single 5, 10, or 20 mg IM dose; HAL^2^)	(24)	5–20	ZIP and HAL were associated with modest QTc increases

Miceli et al., 2010 [19]	3 d single-blind parallel-group	(31 versus 27)	20–30	Mean QTc change (ms):^3^ 1st IV 4.6 (ZIP) and 6.0 (HAL) 2nd IV 12.8 (ZIP) and 14.7 (HAL) No pt had QTc >480 ms QTc ≥450 ms and QTc changes >60 ms: 2 in ZIP (0 in HAL)

Preval et al., 2005 [20]	Naturalistic study (IM ZIP versus first-generation antipsychotics)	(19 versus 80)	20	No patient in the IM ZIP group had QTc >460 ms

Rais et al., 2010 [21]	A 24 h open-label prospective study in geriatric population	(16)	10–20	Mean QTc range in ms: baseline 382.0–429.5 versus trial: 428.1–370.0

Tambyraja and Strawn, 2011 [4]	Case report (70 yo vascular dementia)	(1♀)	20	Patient lost consciousness within 45 min of ZIP IM Pulseless, hypotensive, and poor perfusion signs ECG: bradycardia (30–40 bpm) with 3rd degree block

Li et al., 2010^4^ [16]	3 d double- or single-blind parallel-group RCT (IM ZIP versus HAL^2^)	(16 versus 16)	20–40	No significant QTc prolongations in ZIP or HAL

Witsil et al., 2012 [2]	Case report (history of substance abuse)	(1♂)	20	Baseline ECG showed QT/QTc of 484/475 ms. Palpitations and weakness 45 min after IM ZIP Maximum in repeated ECGs: QT/QTc of 612/517 ms

Yin and Wang, 2012^4^ [22]	3 d open parallel-group RCT (IM ZIP versus HAL^2^)	(30 versus 30)	10–40	Maximum QTc in ZIP: 461 ms.

Zhang et al., 2013^4^ [7]	3 d rater-blind, actively-controlled parallel group multicenter RCT (IM ZIP versus HAL^2^)	(189 versus 187)	10–40	ZIP mean QTc change^3^ in ms: 0.7 (versus −1.6 HAL)^5^ ZIP: 3 pts with QTC ≥450 ms. None with QTC ≥450 ms HAL: 2 pts with QTC ≥480 ms and 1 with QTC ≥500 ms

bpm: beats per minute; d: day; ECG: electrocardiogram; h: hour; HAL: haloperidol; inpts: inpatients; IM: intramuscular;

IV: intravenous; min: minutes; ms: milliseconds; PO: by mouth; pt: patient; RCT: randomized controlled trial; QTc: QTc interval; wk: week; yo: year-old; ZIP: ziprasidone.

^1^Ziprasidone dose in mg/day.

^2^HAL dose ranged from 5 to 20 mg/day in the various studies.

^3^QTc change from baseline to end of treatment.

^4^In Chinese.

^5^Effect size of the mean difference = 0.009. It was calculated using Cohen's method [23].
